# Non-Coding RNA: Sequence-Specific Guide for Chromatin Modification and DNA Damage Signaling

**DOI:** 10.3389/fgene.2015.00320

**Published:** 2015-11-13

**Authors:** Sofia Francia

**Affiliations:** ^1^IFOM – FIRC Institute of Molecular OncologyMilan, Italy; ^2^Istituto di Genetica Molecolare, Consiglio Nazionale delle RicerchePavia, Italy

**Keywords:** non-coding RNA, DNA-damage response, RNA interference, chromatin modulation, transcription

## Abstract

Chromatin conformation shapes the environment in which our genome is transcribed into RNA. Transcription is a source of DNA damage, thus it often occurs concomitantly to DNA damage signaling. Growing amounts of evidence suggest that different types of RNAs can, independently from their protein-coding properties, directly affect chromatin conformation, transcription and splicing, as well as promote the activation of the DNA damage response (DDR) and DNA repair. Therefore, transcription paradoxically functions to both threaten and safeguard genome integrity. On the other hand, DNA damage signaling is known to modulate chromatin to suppress transcription of the surrounding genetic unit. It is thus intriguing to understand how transcription can modulate DDR signaling while, in turn, DDR signaling represses transcription of chromatin around the DNA lesion. An unexpected player in this field is the RNA interference (RNAi) machinery, which play roles in transcription, splicing and chromatin modulation in several organisms. Non-coding RNAs (ncRNAs) and several protein factors involved in the RNAi pathway are well known master regulators of chromatin while only recent reports show their involvement in DDR. Here, we discuss the experimental evidence supporting the idea that ncRNAs act at the genomic loci from which they are transcribed to modulate chromatin, DDR signaling and DNA repair.

## Introduction

Genetic information is transmitted as DNA, yet is functional as RNA in cellular organisms. Genome integrity and as a consequence transcription fidelity is continuously harmed by DNA lesions. The cascade of events that starts with the detection of DNA lesions proceeds through signaling pathways and eventually triggers repair is known as the DNA damage response (DDR; Ciccia and Elledge, 2010). Among the different kinds of lesions, DNA double-strand breaks (DSBs) are the most deleterious and must be accurately repaired. It has been evident for several years that RNA is an important player in the regulation of chromatin and transcription (Holoch and Moazed, 2015) but only recently has RNA been shown to directly participate in preserving genome integrity (Sabin et al., 2013; d’Adda di Fagagna, 2014). This occurs in different ways: not only do RNA transcripts promote DDR signaling when DNA damage arises at their genetic loci (Francia et al., 2012) and guide homology-directed DNA repair (Wei et al., 2012; Gao et al., 2014) but also provide an intact copy of corrupted genetic information to be used as a template for DSB repair (Storici et al., 2007; Keskin et al., 2014). These studies reveal that transcripts can play an active role in preserving the integrity of the genome from which they are generated, raising the paradox that transcription can lead to DNA damage while transcripts are beneficial for efficient DNA damage signaling and repair at the loci from which they are transcribed.

The generation of a DSB causes the appearance of transcripts mapping to sequences flanking the site of damage (Francia et al., 2012; Michalik et al., 2012; Wei et al., 2012) referred to as DNA damage-response RNAs (DDRNAs; Francia et al., 2012) or double-strand break-induced RNAs (diRNAs; Wei et al., 2012). Importantly, transcripts map extremely close to DNA lesion and form also if DNA damage is generated at a locus devoid of promoter sequences and positioned away from other transcription units, excluding the possibility that transcripts are generated passively (Francia et al., 2012). This novel phenomenon appears to be evolutionarily conserved since it has been reported in plants (Wei et al., 2012), insects (Michalik et al., 2012), mouse and human cells (Francia et al., 2012; Gao et al., 2014). However, these findings are contradictory to the more commonly accepted view that sees transcription as an inherently mutagenic process (Bermejo et al., 2012; Kim and Jinks-Robertson, 2012; Hamperl and Cimprich, 2014; Sollier et al., 2014) and DDR activation antagonistic to transcription (Shanbhag et al., 2010). A unifying model that reconciles these contradictory theories is missing.

The use of deep sequencing technology lead to a revolution in our understanding of epigenetic mechanisms behind eukaryotic genome functions by uncovering the existence of ncRNAs which act as master chromatin regulators (Peschansky and Wahlestedt, 2014). DDRNAs are ncRNAs required for activation of the DDR (Francia et al., 2012; Wei et al., 2012), but the mechanism by which they function is still under investigation. Because DDRNAs are active at very low abundance (Francia et al., 2012), a characteristic consistent with other epigenetic regulatory transcripts (Wang et al., 2008; Gupta et al., 2010; Sado and Brockdorff, 2013), it is tempting to speculate that they control DDR activation and DNA repair by modulating chromatin. Here, we discuss how ncRNAs and factors of the RNAi pathway are known to influence chromatin and transcription and we comment about the influence that DDRNAs might have on chromatin during DDR activation and DNA repair.

### Long ncRNAs in Chromatin and Transcription Regulation

Non-coding RNAs are generally classified based on their length: long ncRNAs (lncRNAs) are considered all non-coding transcript longer then 200 base-pairs up to several kilobases; while below this arbitrary threshold ncRNAs are generally referred to as small ncRNAs (sncRNAs). However, this gross classification only defines two overarching categories, each of which includes several different families based on ncRNA biogenesis, localization and function (St Laurent et al., 2015). Apart from the well known big classes of ribosomal RNAs, transfer RNAs, heterogeneous nuclear RNAs, small nucleolar RNAs and ribozymes, several novel lncRNAs have been identified and are often involved in the epigenetic regulation of the eukaryotic genome. Indeed, lncRNAs have the ability to recruit chromatin modifying complexes to defined genomic loci through the concomitant function of protein-binding domains, and sequence specificity (Cech and Steitz, 2014). A paradigm for lncRNA-directed site-specific chromatin modulation comes from the characterization of X-chromosome inactivation (XCI) for dosage compensation. XCI is achieved by the cells through the deposition of H3K27me3 repressive histone marks by the Polycomb repressive complex PRC2, which is recruited to only one X chromosome via a direct interaction with lncRNA transcript from the X inactive-specific transcript (Xist) locus (Plath et al., 2003; Galupa and Heard, 2015). Acting locally as a sequence-specific scaffold, *Xist* lncRNA is active at a very low copy number per cells (Zhao et al., 2008). Intriguingly, few studies reported the interactions of *Xist* lncRNA with BRCA1, a DNA repair protein required for homologous recombination (HR) which has also been proposed to participate in XCI (Ganesan et al., 2002, 2004). Nevertheless, the putative involvement of BRCA1 in XCI have been challenged in other reports (Xiao et al., 2007). Similarly to *Xist*, other chromatin bound ncRNA act *in cis* as few copies per cell to repress transcription. One example is the *ncRNA_CCND1_* whose expression is induced upon DNA damage at the 5′ regulatory regions of CCND1. *ncRNA_CCND1_* allosterically interacts with the RNA binding protein FUS/TLS and represses the expression of CCND1 by inhibiting the activity of p300 histone acetyltransferase locally (Wang et al., 2008). A number of other lncRNAs are induced upon DNA damage, often in a TP53-dependent manner. Two examples are the long intergenic non-coding RNA-p21 (*lincRNA-p21*; Huarte et al., 2010) and the lncRNA named p21 associated ncRNA DNA damage activated (*PANDA*; Hung et al., 2011), both transcribed upstream of the cell cycle regulator p21. *LincRNAp21* binds to the transcription factor hnRNP-K and controls the proper silencing of TP53-repressed genes (Huarte et al., 2010) while *PANDA* binds to NF-YA, PRC1 and PRC2 to modulate the expression of pro-apoptotic and senescence genes upon DNA damage (Puvvula et al., 2014; Zhang and Peng, 2015).

Long non-coding RNA with antisense orientation control the expression of complementary transcripts *in cis*. Intriguing examples are the *p15AS* (p15 antisense transcript), which is expressed in antisense orientation to the cyclin-dependent kinase inhibitor p15 and controls its silencing by heterochromatin formation in a DICER-dependent manner (Yu et al., 2008), and the KCNQ1 antisense transcripts (*KCNQ1ot1*) which represses KCNQ1 by recruiting the H3K9 histone methyltransferase G9a and the PRC2 complex increasing the level of H3K9me3 and H3K27me3 *in cis* to its locus (Pandey et al., 2008). Similarly, the monoallelically expressed ncRNA *AIR* represses Slc22a3, Slc22a2, and Igf2r genes *in cis* by interacting and recruiting G9a (Nagano et al., 2008).

However, not all ncRNAs act by modulating chromatin *in cis*. The long intergenic ncRNA HOX transcript antisense RNA (*HOTAIR*), which originates from the HOXC locus, represses transcription of the HOXD locus *in trans* by recruiting PRC2 (Rinn et al., 2007; Gupta et al., 2010; Spitale et al., 2011). *HOTAIR* can directly interact with both PRC2 and the LSD1/CoREST/REST histone de-methylase complex, thereby inactivating transcription at target sites by coupling H3K27 methylation with de-methylation of H3K4 (Tsai et al., 2010). *HOTAIR* misregulation has been observed in a variety of cancers and might affect the expression of genes involved in apoptosis, growth and metastasis (Yu and Li, 2015). Another lncRNA relevant for cancer is *ANRIL* that, by interacting with the PRC1-component CBX7, contributes to repress the INK4b/ARF/INK4a locus and therefore limits senescence (Yap et al., 2010).

Non-coding RNA scaffolds not only induce repressive chromatin conformation but also positively influence transcription. This is the case of *HOTTIP*, a lincRNA transcribed from the 5′ tip of the HOXA locus that, by targeting WDR5/MLL complexes across HOXA and driving histone H3K4 trimethylation, coordinates the activation of HOXA genes at specific timing *in vivo* (Wang et al., 2011a). Chromosomal looping and high order structure necessary for gene activation have been proposed to be guided by *HOTTIP* lncRNA (Wang et al., 2011a), similar to what occurs for the ncRNAs with enhancer functions (eRNAs), other activating lncRNAs whose transcription stimulates the expression of neighboring genes (Orom et al., 2010; Lai et al., 2013; Andersson et al., 2014). Another way in which RNA changes the architecture of chromatin is by binding multiple proteins, as demonstrated by the *PCGEM*1 lncRNA that interacts with the androgen receptor (AR) and the activating chromatin effector *Pygopus* homolog 2 (Pygo2), thereby enhancing selective looping of AR-bound enhancers to target gene promoters (Yang et al., 2013).

Overall, a unifying theme in ncRNA-directed chromatin modification is the use of transcripts as a scaffold to achieve locus specific chromatin modification a well characterized mechanism that raises the apparent paradox that RNA-guided transcriptional silencing requires on-going transcription of the same locus.

An additional mechanism proposed for the targeting of these lncRNA to the correct locus is via lncRNA:DNA triplex formation. One example is the recently identified *MEG3* lncRNA which represses genes of TGF-beta pathway by recruiting EZH2 via RNA:DNA triplex formation (Mondal et al., 2015). Previously lncRNA:DNA triplex formation was proposed in the case of the promoter associated *pRNA*, ncRNA complementary to the rDNA promoter that, by recruiting the DNA methyltransferase DNMT3b, mediates *de novo* CpG methylation and silencing of rRNA gene promoters (Schmitz et al., 2010).

In **Table 1** are listed the lncRNAs discussed.

**Table 1:** List of lncRNAs discussed in the text. For each of them the interacting protein-complexes, the epigenetic modification and/or the related functional outcome are specified.

lncRNA	Chromatin modifying complex	Epigenetic modification	Functional outcome
Xist	PRC2 complex	H3K27me3	Transcriptional repression of inactive X chromosome
HOTAIR	PRC2 LSD1	H3K27me H3K4 de-methyl	Transcriptional repression of HOX genes
ANRIL	CBX7/PRC1	H3K27me3	Transcriptional repression of INK4 locus
KCNQ1ot1	G9a PRC2	H3K9me3 H3K27me3	Transcriptional repression of KCNQ1 gene
AIR	G9a	H3K9me3	Transcriptional repression of Slc22a3, Slc22a2, and Igf2r genes
p15AS	–	H3K9me3 H3K4de-methyl	Transcriptional repression of p15
LincRNAp21	hnRNPK	–	Transcriptional repression of TP53-repressed genes
PANDA	NF-YA PRC1	–	Transcriptional repression of pro-apoptotic and senescence genes
ncRNA_CCND1_	FUS/TLS	–	Transcriptional repression of CCND1
pRNA	DNMT3b	DNA methylation	Transcriptional repression of rDNA
HOTTIP	WDR5/MLL	H3K4me3	Transcription activation of HOX genes
PCGEM1	Androgen receptor PYGO2	Chromatin looping	Enhanced transcription of androgen receptor-regulated genes
eRNAs	Mediator	–	Enhanced transcription of target genes


### Small Non-coding RNA and the RNA Interference Machinery in DDR and Chromatin Modulation

Small non-coding RNAs (sncRNAs) are also divided in several different classes. Among a number of sncRNAs families, the more characterized are microRNAs (miRNAs or miR): DICER- and DROSHA-dependent sncRNAs which control the expression of more than 30% of human coding transcripts both at post-transcriptional and transcriptional level (Pasquinelli, 2015). Certainly, also DNA damage is a stimulus that results in altered expression of several miRNA families (Wouters et al., 2011). In particular, a group of miRNA promoters are targeted by the DDR effector and transcription factors TP53. Most relevant examples are the miR34a-c family and the miR29 family (Hermeking, 2012) which are induced in cells exposed to different genotoxic stress, and miR200c which increases following oxidative stress and triggers apoptosis and senescence by targeting ZEB1 (Magenta et al., 2011). The miR34a-c family represses the mRNA transcripts of several genes involved in cell proliferation and survival, such as BCL2, Cyclin D1 and E2 and cyclin-dependent kinases (CDK) 4 and 6, and therefore controls both apoptosis and senescence (Chang et al., 2007; Raver-Shapira et al., 2007; Tazawa et al., 2007; Yamazaki et al., 2012). The TP53 dependent up-regulation of the miR29 family, which represses the TP53 inhibitor Wip1 phosphatase, ultimately leads to TP53 induction and contributes to a positive feed-back loop during aging or chronic DDR activation (Ugalde et al., 2011). Other miRNAs induced upon DNA damage in a TP53-dependent manner and relevant for cancer are miR192, miR194, and miR215 (Braun et al., 2008). Intriguingly, mutant TP53 expressed in cancer not only loses its oncosuppressive properties but also regulates the expression of a different cohort of miRNAs responsible for its oncogenic activity (Liao et al., 2014). Moreover, TAp63, another member of the TP53 family, binds to DICER gene promoter and *trans*-activates it, directly controlling the processing of all miRNAs (Su et al., 2010).

DNA damage and DDR activation can also regulate miRNA expression by modulating the miRNA processing at maturation steps. Following DNA damage, the main DDR kinase Ataxia telangiectasia mutated ATM phosphorylates the KH-type splicing regulatory protein KSRP (Zhang et al., 2011), a multifunctional RNA-binding protein that interacts with both DROSHA and DICER (Trabucchi et al., 2009, 2011). KSRP was reported to interact with guanosine-rich regions in the terminal loop of miRNA precursors (pre-miRNA), and its ATM-dependent phosphorylation significantly enhanced the maturation of a cohort of pri-miRNA including pri-let 7, whereas KSRP mutations in the ATM-dependent phosphorylation sites impair its miRNA-regulating activity (Trabucchi et al., 2009, 2011; Gherzi et al., 2014). It was also shown that ATM dependent phosphorylation of exportin-5, which mediates the nuclear export of miRNAs, enhances its interaction with nuclear pores after DNA damage, defining an additional level of miRNA modulation by DNA-damage signaling (Wan et al., 2013).

Transcripts coding for key factors involved in DDR and chromatin modulation are also targeted by specific miRNAs at the post-transcriptional level following DNA damage. For example, one of the earliest events in the DDR is the ATM-dependent phosphorylation of the histone variant H2AX (Ciccia and Elledge, 2010), the coding mRNA of which is targeted by both miR24 (Lal et al., 2009; Brunner et al., 2012) and miR138 (Wang et al., 2011b; Yang et al., 2015a). Indeed, the overexpression of these miRNAs increases sensitivity to DNA damaging agents (Lal et al., 2009). ATM mRNA itself is also targeted by some miRNAs up-regulated in cancer, such as the N-myc-induced miR421 (Hu et al., 2010; Mansour et al., 2013), but also miR101 (Yan et al., 2010) and miR100 (Ng et al., 2010) whose overexpression enhances radiosensitivity by down-regulating ATM. Similarly, miR182 down regulates BRCA1 levels in human breast cancer cells, thus impeding repair by HR and sensitizing cell to treatment with PARP inhibitor and ionizing radiation (Moskwa et al., 2011; Krishnan et al., 2013).

However, a different group of miRNA has a positive impact on DDR activation. The recently described miR339-5p, and miR542-3p, indirectly stabilize TP53 by repressing MDM2 (Wang et al., 2014; Zhang et al., 2015). Overall, there is complex network of mutual modulations between DDR factors, DDR activation and miRNA biogenesis.

Transcripts originating from DNA breaks are processed by two ribonucleases of the RNAi machinery DROSHA and DICER, into sncRNAs (Francia et al., 2012; Michalik et al., 2012; Wei et al., 2012) and have been proposed to act in HR associated with the RNA-induced silencing complex (RISC) component ARGONAUTE 2 (AGO 2; Gao et al., 2014). RNA-processing enzymes have been linked to DDR activation in yeast as well (Manfrini et al., 2015). In agreement to the well-known function of RNAi in regulating gene expression by post-transcriptional gene silencing, DDRNAs have been shown to act as endogenous siRNA against truncated transcripts accumulating as a consequence of lesion generation (Michalik et al., 2012). However, sncRNA processed by the RNAi pathway are also known to silence gene expression at transcriptional level by modulating chromatin (Guang et al., 2008; Fagegaltier et al., 2009; Cernilogar et al., 2011). Indeed sncRNAs can act as guiding molecules for chromatin modifier enzymes in lower organisms (Fukagawa et al., 2004; Buhler et al., 2006; Castel and Martienssen, 2013; Keller et al., 2013). In mammals, RNAi factors such as DICER and AGO, historically considered merely cytoplasmatic, have been shown to localize in the nucleus of mammalian cells while maintaining activity (Janowski et al., 2006; Meister, 2013; Du Toit, 2014; Gagnon et al., 2014; Gao et al., 2014; White et al., 2014) and DICER-dependent, sequence-specific sncRNAs among which some miRNAs have been shown to control transcription at chromatin level in different contexts (Janowski et al., 2006; Kim et al., 2006; Gonzalez et al., 2008). This process is even enhanced in senescent cells, likely to suppress pro-proliferative genes (Benhamed et al., 2012). SncRNA-dependent chromatin modulation in mammals also occurs during deposition of heterochromatin at repetitive sequences (Giles et al., 2010) such as telomeres (Cao et al., 2009), centromeres (Halic and Moazed, 2010; Marasovic et al., 2013) and ribosomal DNA (Sinkkonen et al., 2010), as well as gene termination sites (Skourti-Stathaki et al., 2014). DICER-dependent sncRNAs associated with AGO1 and AGO2 have also been shown to modulate RNA polymerase II (RNAPII) elongation rate in mammalian cells by locally inducing chromatin compaction, thereby influencing splicing choices (Ameyar-Zazoua et al., 2012). A similar effect can be obtained at specific splicing sites via transfection of exogenous siRNA (Allo et al., 2009).

Although in all of these contexts sncRNAs recruit chromatin repressive complexes that induce silencing of target loci, DICER-dependent sncRNAs have also been proposed to play the opposite role of restraining the spreading of constitutive heterochromatin at the boundaries of centromeres (Keller et al., 2013) suggesting that their function might not be limited to heterochromatin formation and transcriptional silencing. Indeed, sequence-specific RNAs, often in complex with AGO2, have been show to map to transcriptional start sites (TSS-RNA) in human (Zamudio et al., 2014), mouse, chicken and fruit flies (Chitwood and Timmermans, 2010; Czech and Hannon, 2011) and small promoter-associated transcripts (PROMPTs) predicted to activate transcription through changes in chromatin structure have been described (Preker et al., 2008; Seila et al., 2008). Also, sncRNA-AGO2 complexes were shown to control transcription through reduction of H3K9 levels at sequence-specific promoter of a target gene, thereby inducing its activation (Li et al., 2006).

Increasing amounts of evidence also indicate that RNAi protein factors regulate gene expression co-transcriptionally through interaction with the transcriptional machinery (Castel and Martienssen, 2013). In *Drosophila melanogaster* Dicer 2 and Ago 2 interact with RNAPII and are required for positioning the transcription machinery on gene promoters in euchromatin (Cernilogar et al., 2011), while in mammals AGO1 associates with RNAPII and induces gene silencing at siRNA-targeted gene promoters (Kim et al., 2006). DROSHA and the DROSHA-containing complex known as microprocessor, have recently been proposed to control gene expression co-transcriptionally in two different ways: first acting at gene promoters by binding to hairpins of nascent transcripts, thus stabilizing them (Gromak et al., 2013), and second by cleaving stem-loops present in specific transcripts to induce transcription termination (Dhir et al., 2015). At sites of DNA damage, AGO2-associated sncRNAs influence the DNA repair pathway by recruiting the HR-factor Rad51 to actively transcribed regions (Gao et al., 2014). This is in agreement with the observation that transcriptionally active chromatin is preferentially repaired by HR (Aymard et al., 2014). Overall, several different examples exist of sequence-specific and RNAi-dependent sncRNAs acting at the level of chromatin in different organisms.

### Endogenous Sources of Long Double-stranded RNA, Precursors of sncRNAs

SncRNAs mostly act as single stranded RNA (ssRNA) molecules, however, DICER is able to cleave only double stranded RNA (dsRNA) precursors (Holoch and Moazed, 2015). How such longer double stranded precursors are generated in different contexts is often unclear. The accepted model of miRNA biogenesis in which a longer ssRNA is folded into a stem-loop secondary structure (Kim et al., 2009) does not always apply to non-miRNA loci. A recently proposed source of dsRNA precursors is oppositely oriented transcription of two independent genetic unit, leading to the formation of partially complementary transcripts able to anneal with each other and form long dsRNA molecules (Gullerova and Proudfoot, 2012; Skourti-Stathaki et al., 2014). Increasing evidence points to the fact that dsRNA generated by overlapping transcripts form relatively frequently *in vivo*. In fact, DICER inactivation leads to increased levels of dsRNAs and consequent activation of the interferon response, in agreement with the idea that transcripts able to form long dsRNAs are fairly abundant and are indeed substrate for DICER processing (White et al., 2014). In support of this notion, overlapping, inversely oriented transcripts originating at regions with high gene density, as well as bidirectional transcription at neighboring transcription units, are not uncommon in the genome of vertebrates (Adachi and Lieber, 2002; Okamura et al., 2008; Orekhova and Rubtsov, 2013). It has been proposed that a common feature of promoters in eukaryotic organisms is bidirectionality (Seila et al., 2009). However, this model is still controversial and other studies have proposed instead that promoters are intrinsically uni-directional (Duttke et al., 2015). An example of widespread and ubiquitous antisense transcripts is the recently characterized class of Natural Antisense Transcripts (NATs), regulatory RNA molecules influencing the expression of the complementary gene (Faghihi and Wahlestedt, 2009; Khorkova et al., 2014). These antisense transcripts may have been largely elusive because they are unstable and rapidly processed, similar to what happens with the recently identified nascent transcripts generated by divergent transcription initiation at promoter and enhancer (Weingarten-Gabbay and Segal, 2014).

**FIGURE 1:**
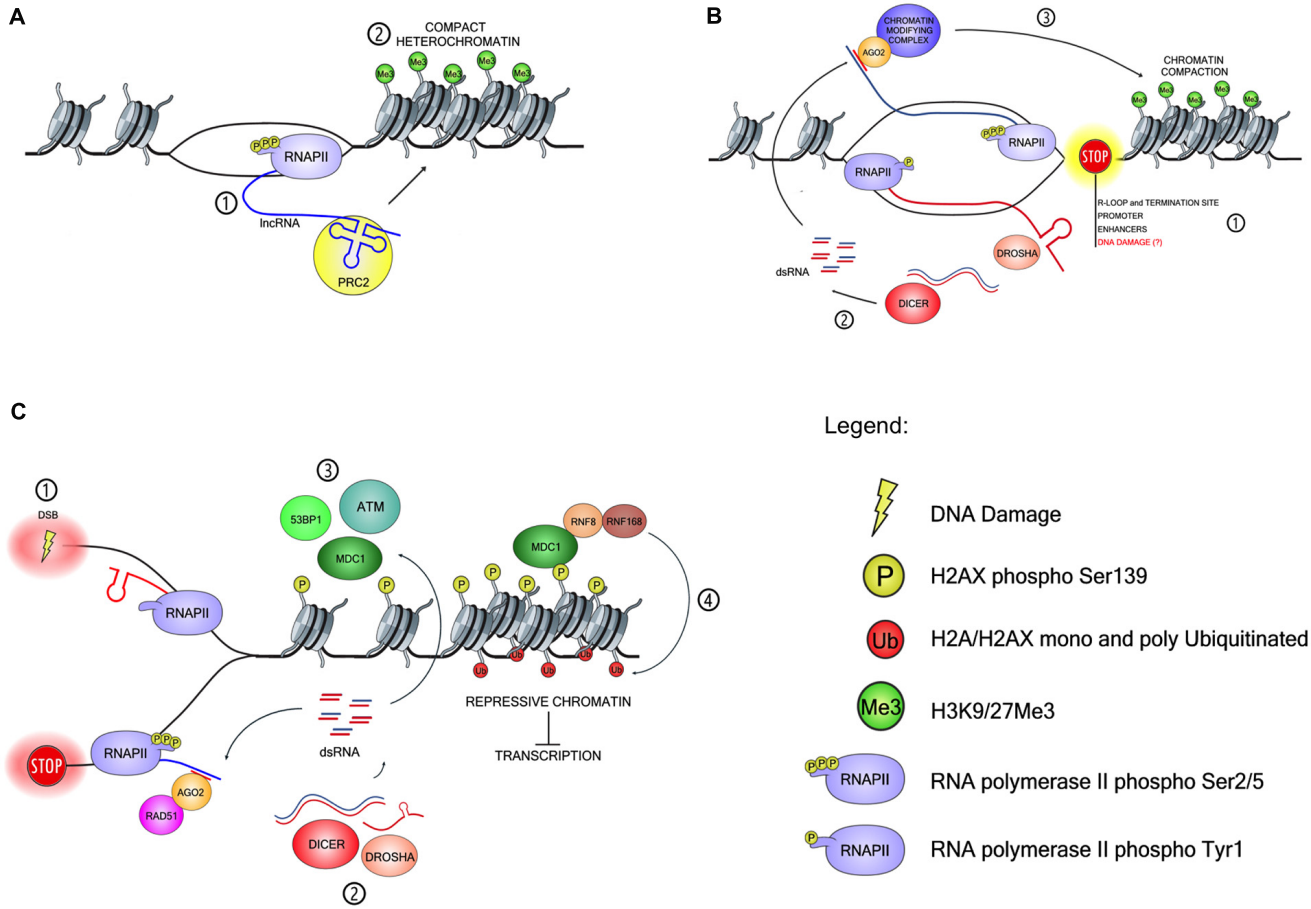
**Models of ncRNA-mediated site-specific chromatin modification and DDR modulation. (A)** Schematic representation of long ncRNA (lncRNA) in transcriptional repression. (1) RNAPII dependent transcripts fold in protein-binding domains and (2) recruit histone-modifying complexes such as PRC2 to chromatin *in cis.* **(B)** Schematic model of small ncRNAs (sncRNAs) generation and chromatin modulation at RNAPII pausing sites. (1) Pausing of RNAPII is induced in physiological condition at promoter and enhancer sequences and at transcription-termination sites prone to form R-loops, but could also occur in the presence of DNA lesions. (2) RNAPII pausing stimulates the loading of another RNAPII in opposite orientation on the complementary DNA template and generates antisense transcripts. DsRNA precursors are then processed by the RNAi-machinery into sncRNA. (3) ARGONAUTE 2 (AGO2) forms a complex with such sequence-specific sncRNA and guide chromatin-modifying enzymes to the pausing site via nascent RNA:sncRNA interaction. **(C)** Schematic model for sncRNAs generation and function at site of DNA damage. (1) Double-strand break (DSB) generation induces stalling of RNAPII and synthesis of partially complementary transcripts. (2) Stem-loop like secondary structures of the transcripts or long dsRNAs precursors are processed by DROSHA and DICER into sncRNA, DDRNA. (3) DDRNA stimulates DDR activation via an unknown mechanism. Initially, DDR activation leads to chromatin de-compaction, which may increase transcription and favors ATM activation. In turn, ATM induces mono- and poly-ubiquitination of H2A/H2AX by RNF8/RNF168 ubiquitin ligases, consequent chromatin compaction and transcriptional silencing (4). Chromatin compaction might promote reciprocal interactions between DDR proteins through their increased density, further boosting DDR activation in the absence of additional transcription.

Another interesting source of dsRNA has been proposed at human promoters where an engaged RNAPII is loaded in an opposite orientation respect to the gene and synthesizes complementary RNAs of few hundred bases but does not elongate beyond the promoter (Core and Lis, 2008; Core et al., 2008). In support of this model, phosphorylation of Tyrosine 1 on the RNAPII C-terminal domain (CTD) has been associated with antisense transcription at promoters and enhancers of mammalian cells (Descostes et al., 2014). Moreover, at RNAPII pausing sites upstream to promoters dsRNAs are known to accumulate (Flynn et al., 2011). Therefore, it has been proposed that a paused promoter-proximal RNAPII can synthesize both RNA strands that then anneal forming a dsRNA, which can be processed by DICER (Du Toit, 2014). Bound to AGO, these sncRNAs are believed to influence the local chromatin conformation, thus fine-tuning the switch between paused and elongating RNAPII. A similar mechanism has been described at transcription termination sites where RNAPII pausing is caused by R-loops (Skourti-Stathaki et al., 2014) and sncRNAs associated with AGO proteins are believed to recruit chromatin modifier enzymes and increase local chromatin compaction. Likewise, RNAPII is known to pause at alternative splice sites (Allo et al., 2009; Saint-Andre et al., 2011) where RNAi-dependent sncRNAs accumulate, further suggesting that paused RNAPII may generate antisense transcription and therefore dsRNA molecules. It is therefore plausible that in the physiological context of gene transcription, DNA damage and DDR signaling may induce RNAPII pausing and consequent synthesis of complementary transcripts. Once annealed, these transcripts may provide a dsRNA precursor for DICER-dependent DDRNA biogenesis that, in turn, may stimulate DDR activation and guide-chromatin modulation (see **Figure 1** for a schematic representation).

In plants and yeasts an additional source of dsRNA precursors is provided by RNA dependent RNA polymerases (RdRP), able to synthesize the complementary strand of a single stranded RNA template (Ahlquist, 2002). Most higher eukaryotes also encode putative RdRPs, but in mammals RdRP activity associated with RNAi has remained elusive (Maida and Masutomi, 2011). Recently, it has been proposed that mammalian RNAPII retains RdRP activity similar to that of budding yeast RNAPII (Lehmann et al., 2007) and is involved in generating complementary strands of non-coding transcripts to target them for degradation (Wagner et al., 2013). A similar RdRP activity has been postulated for human telomerase TERT, which was proposed to produce telomeric dsRNAs then processed into sncRNA in a DICER-dependent manner (Maida et al., 2009; Maida and Masutomi, 2011) suggesting that TERT may contribute to amplifying sncRNA biogenesis from telomeric sequences. However, the putative RdRP activity of TERT is still under debate (Martinez and Blasco, 2011). At site of DNA damage, diRNA biogenesis requires RdRP activity in plants (Wei et al., 2012), suggesting that a residual RdRP activity of RNAPII could be involved in DDRNA biogenesis in mammalian cells too.

### Chromatin Compaction and Relaxation at Sites of DNA Damage

Both condensation and relaxation have been show to occur at damaged chromatin. Recently, it has been shown that chromatin compaction after DNA damage is a secondary event that follows an initial chromatin relaxation (Burgess et al., 2014). Adjacent to DSBs, local chromatin de-condensation as well as histone reorganization and eviction have been previously observed in mammalian cells (Kruhlak et al., 2006; Ziv et al., 2006; Berkovich et al., 2007; Price and D’Andrea, 2013). It is also well established that important chromatin remodeling complexes such as SWI/SNF (Smith-Roe et al., 2015), CHD (Stanley et al., 2013), INO80 (Kashiwaba et al., 2010), SMARCA5 (Smeenk et al., 2013), ISWI (Aydin et al., 2014) and the poly (ADP-ribosyl)ation dependent, nucleosome-repositioning enzyme ALC1 (Ahel et al., 2009), are recruited to DSBs. The putative purpose of this recruitment is to remove nucleosomes from damaged DNA, shift back the position of nucleosomes and substitute histone variants (Panier and Durocher, 2013; Price and D’Andrea, 2013; Smeenk et al., 2013). Moreover, well-known histone marks characteristic of open chromatin are deposited after DNA damage in a DDR-dependent manner: histones H2A and H4 acetylation by the histone acetyl transferases TIP60 (Sun et al., 2009) and MOF (Sharma et al., 2010) and the monoubiquitination of histone H2B by RNF20/RNF40 (Miller and Jackson, 2012), have been extensively shown to be deposited at site of DNA damage. Interestingly, both MOF (Akhtar and Becker, 2000) and RNF20/40 are well known transcription activators (Shiloh et al., 2011) that also modulate chromatin de-condensation at transcribed regions, suggesting that similar chromatin modifications might be required for DDR activation.

A number of other processes that promote chromatin relaxation and are required for proper DDR activation have been reported, such as the mobilization of heterochromatin-associated factors HP1 and KAP-1 (Ziv et al., 2006; Ayoub et al., 2008; Noon et al., 2010; Bolderson et al., 2012) away from damaged loci and the re-localization of DNA breaks at the periphery of heterochromatin or inside euchromatin (Chiolo et al., 2011; Jakob et al., 2011). Collectively, these reports prove that active chromatin de-compaction at sites of DNA damage is a controlled process, rather than a side effect given by DNA breakage, raising questions about the possible mechanism by which both chromatin relaxation and re-compaction are modulated by DDR. Both events seem to play a functional role in DDR since the loss of the initial chromatin de-compaction but also the induction of persistent de-compaction on damaged chromatin impair DDR signaling (Burgess et al., 2014) and repair (Kakarougkas et al., 2014; Kalousi et al., 2015). On the other hand, forced chromatin compaction can also induce ATM signaling in the absence of physical DNA damage (Kaidi and Jackson, 2013). Overall, not only chromatin compaction but also local chromatin relaxation is intimately associated with DDR signaling at damaged chromatin. Antagonistic signals during DDR signaling have been implicated in providing boundaries to its spread along chromatin (Panier and Durocher, 2013). Thus, the fact that these two apparently contradictory phenomena coexist at site of damage may suggest that they finely tune each other.

During DDR activation two consecutive steps can be distinguished: the first consists of a primary, direct recognition of DNA lesions by DDR sensors, while the second consists of an amplification phase mediated by chromatin modifications that recruit and retain DDR mediators and kinases in close proximity, promoting their interaction (Fernandez-Capetillo et al., 2003; Bekker-Jensen and Mailand, 2010). A distinct, yet not alternative scenario envisages that the initial recognition of a DNA lesion requires chromatin de-compaction to allow the binding of DDR-sensors to damaged DNA, while the following DDR signal amplification might be enhanced by compaction, which increase the local signal and favors the reciprocal interaction among DDR proteins. Additionally, the initial chromatin de-compaction could indeed promote local transcription that generates RNA precursors for DDRNA biogenesis, in turn required to boost DDR signaling and thus repair (**Figure 1**), (Francia et al., 2012; d’Adda di Fagagna, 2014).

### The Intricate Relationship between DDR Signaling and Transcription

A genomic locus is repaired more efficiently when actively transcribed than when kept silent (Goodarzi et al., 2008; Chaurasia et al., 2012; Fong et al., 2013), an observation suggesting that on-going transcription can play a positive role in DNA repair. Along the same line, RNAPII and the transcription machinery have been proposed to act as genome scanner, detecting and signaling the presence of DNA damage (Lindsey-Boltz and Sancar, 2007; Montecucco and Biamonti, 2013; Winsor et al., 2013). In this scenario, the recent understanding that most of our genome is pervasively transcribed at a basal level (Kapranov et al., 2007; Berretta and Morillon, 2009; Clark et al., 2011) emphasizes how prominent the role of transcribing RNAPII could be in the detection of damaged DNA and activation of consequent repair. More intriguingly, inhibition of RNAPII elongation by specific antibodies induces the ATM dependent phosphorylation of the tumor protein TP53 in the absence of DNA damage and in a replication-independent manner (Ljungman et al., 2001; Derheimer et al., 2007), highlighting that DDR might be activated upon inhibition of RNAPII elongation.

One related fascinating topic is how transcription is modulated around DNA breaks. It has been shown that DNA breaks can cause transcription inhibition of adjacent genes via two complementary mechanisms: by surrounding the damaged locus with repressive chromatin modifications and/or by excluding elongating RNAPII from damaged sites. An elegant study in a conveniently engineered cellular system shows that a cluster of DSBs generated by a restriction enzyme at a repetitive locus stimulates DDR-mediated chromatin silencing that spreads for kilobases from the damaged locus (Shanbhag et al., 2010). It is now well established that DNA damage-induced ATM activation recruits Ring Finger protein 8 (RNF8) and RNF168 E3-ubiquitin ligases that ubiquitinate histones H2A/H2AX in chromatin surrounding the breaks (Jackson and Durocher, 2013; Brown and Jackson, 2015). This event is believed to cause chromatin compaction and has been linked to repression of transcription units positioned a few kilobases downstream from the cluster of DSBs (Shanbhag et al., 2010). Importantly, transcription inhibition induced by DSB generation can be reversed by ATM inhibition or RNF8 and RNF168 co-depletion while still in the presence of DNA damage (Shanbhag et al., 2010), suggesting that transcription is not physically impeded by the DNA lesions itself, but is instead actively controlled by DDR-activation. In the same cellular system it has been shown that the chromatin remodeling complex Polybromo BRG1 (Brahma Related Gene 1) Associated Factor (PBAF) is phosphorylated by ATM and, together with the Polycomb repressive complexes PRC1 and PRC2, is required for transcription repression *in cis* to the DSB cluster (Kakarougkas et al., 2014).

Interestingly, the down-regulation of transcriptional units due to a nearby damaged locus correlates with a reduced presence of the elongating RNAPII isoform but not of total RNAPII at the γH2AX-positive domain (Shanbhag et al., 2010), suggesting that RNAPII might be initially pausing at damaged chromatin rather than being excluded from it. This hypothesis is in line with the recent observation that cells treated with DSB-inducing agents show an increase of RNAPII association with chromatin, rather than a decrease (Britton et al., 2014). Several other reports suggest the opposite, showing the exclusion of RNAPII from γH2AX positive chromatin when laser micro-irradiation is used to deliver a high dose of DNA damage in a confined area (Chou et al., 2010; Miller et al., 2010; Seiler et al., 2011; Polo et al., 2012).

Repression of transcription adjacent to a DNA lesion is also suggested by the fact that several heterochromatin markers and heterochromatinization-inducing enzymes are present at sites of DSBs. Some examples are HP1 (Baldeyron et al., 2011), the histone methyltransferases SUV3-9 and PRDM2, responsible for the deposition of the H3K9me3 (Ayrapetov et al., 2014), the histone deacetylases HDAC1 and HDAC2 (Luijsterburg et al., 2009; Zarebski et al., 2009; Miller et al., 2010) and the histone methyltransferase MMSET (Pei et al., 2011). Moreover, chromatin-repressive complexes, such as PBAF, Polycomb and the Nucleosome Remodeling Deacetylase NuRD complex, associate with damaged chromatin (Chou et al., 2010; Kakarougkas et al., 2014; Leung et al., 2014). Overall, it is well established that DDR activation induces a signaling pathway that results in repression of adjacent genes, a phenomenon that might be useful to counteract the accumulation of corrupted or truncated transcripts and to avoid collisions between transcription and repair intermediates.

Conversely, it has been proposed that when a single DSB is generated inside a gene, RNAPII elongation rate is only transiently arrested by DNAPKcs-dependent DDR signaling (Pankotai et al., 2012). DNA-PKcs inhibition allows transcription elongation to take place, regardless of the presence of the break, and possibly even across the break (Pankotai et al., 2012). This provocative interpretation is in line with the *in vitro* observation that RNA polymerases can bypass different types of DNA lesions, including DNA breaks, by mis-incorporating ribonucleotides, a process known as transcriptional mutagenesis (Doetsch, 2002; Saxowsky and Doetsch, 2006; Pankotai and Soutoglou, 2013; Xu et al., 2014). Thus, a possible model to reconcile these contradictory observations predicts that DDR activation modifies chromatin to induce transcriptional repression at heavily damaged chromosomes, while a single DNA break causes only a transient RNAPII pausing, thus enabling a rapid restart after DNA repair.

### RNAi Pathway and Innate Immunity against Invading Genetic Elements

sncRNAs are known to take part in other essential events linking chromatin and transcription modulation with genome instability, such as genome defense from invading nucleic acids and transposable elements (TE; Fagegaltier et al., 2009; Cam, 2010). In this context, RNAi-dependent sncRNAs guide the deposition of repressive histone marks at invading genetic element through base pairing with nascent transcripts.

It is easy to imagine that complementary transcripts might originate from the expression of repetitive elements such as Long Interspersed Nuclear Elements (LINE) and Short Interspersed Nuclear Elements (SINE), which derive from retrotransposons and Alu repeats. LINE and SINE are present in millions of copies in our genome occupying respectively 17% and 11% of it. Indeed, by inducing transcriptional gene silencing of repetitive sequences, RNAi represents one of the main forms of innate immunity against viruses and invading genetic elements in all organisms (Obbard et al., 2009). From archaebacteria, the RNAi related CRISPR/Cas immune pathway exploits an RNA-guide to direct sequence specific DNA cleavage against invading phages or plasmids (Rimer et al., 2014). It has been recently demonstrated that DNA target recognition involves its transcription and that cleavage by Cas can occur both on DNA and RNA complementary molecules in the case of Type III CRISPR-Cas system (Samai et al., 2015).

In plants, RNAi is a major source of immunity functioning through induction of exogenous DNA silencing via methylation (Kim and Zilberman, 2014), while in animals the evolution of a protein-based adaptive immune response has partially reduced the need of antiviral RNAi activity. Still, TEs and viruses have invaded and profoundly shaped the mammalian genome (Buckley and Lis, 2014). It has been estimated that almost 40% of our genome is composed of invading genetic elements such as integrated viruses and TEs, which, once activated, are potent inducers of DNA damage (Goodier and Kazazian, 2008). In human cells, DICER has been shown to play an important role in counteracting Alu-dependent dsRNA accumulation and toxicity (Kaneko et al., 2011).

RNA-mediated control of transposon activation is particularly active in the mammalian germ line where a devoted class of small-interfering RNA, PIWI-interacting RNA (piRNA), has been linked to both epigenetic and post-transcriptional gene silencing of retrotransposons (Siomi et al., 2011; Castel and Martienssen, 2013). piRNAs are not expressed in somatic cells but are aberrantly re-expressed in cancer cells, suggesting that TE could be reactivated by the large amount of DNA damage experienced by cancer cells and in turn cause additional deleterious mutations promoting cellular transformation (Cheng et al., 2011). To protect themselves, viruses and TEs suppress RNAi and try to escape RNAi control by evolving rapidly. It has been proposed that genes encoding for the RNAi factors have co-evolved quickly with the viruses that they counteract (Obbard et al., 2006).

### Quelling: An Ancient Link between RNAi and Genome Instability

Another interesting phenomenon linking RNAi-guided gene silencing and control of genome stability is quelling in *Neurospora crassa* (Pickford et al., 2002; Fulci and Macino, 2007; Lee et al., 2009) a post transcriptional gene silencing triggered by multiple copies of a transgene, similar to co-suppression in *Caenorhabditis elegans* (Pickford et al., 2002; Fulci and Macino, 2007; Lee et al., 2009). During quelling, ‘abortive’ transcripts from the transgene are synthesized by the DNA-dependent RNA polymerase QDE-1 and amplified by the RdRP activity of QDE-1 itself (Cogoni and Macino, 1999a,b). DsRNA is then processed by dicing enzymes, generating sncRNAs necessary for post transcriptional gene silencing of the transgene itself (Catalanotto et al., 2004). Both sncRNAs and the Argonaute-related protein, QDE-2, whose mutation yields DNA damage sensitivity, are overexpressed upon DNA damage (Lee et al., 2009), a fact suggesting that quelling might be involved in DDRNAs biogenesis in *N. crassa*. Similarly to what occur during quelling, DNA breaks in endogenous epetitive sequences might induce the expression of complementary transcript processed into sncRNAs, which in turn stimulate repair and silencing of the repetitive DNA (Yang et al., 2015b).

## Conclusion

It seems that sncRNAs play an evolutionarily conserved role in both chromatin dynamics and genome stability. Thus, the discoveries of DDRNAs and diRNAs highlight the existence of a consistent, yet previously unknown, sncRNA-mediated layer in the regulation of DDR signaling and DNA repair. This discovery raises the possibility of novel approaches in designing chemical or other pharmacological molecules that might act on, or through, the DDR pathway in hopes of identifying novel strategies for disease treatment (Pearl et al., 2015). The idea that DDRNAs control DDR activation is likely interconnected with the fact that such sncRNAs may also modulate chromatin around damaged DNA. However, the exact role played by DDRNA in controlling chromatin conformation is still poorly defined and needs further investigation.

Given the involvement of DDR in a number of physiological and pathological processes such as immunodeficiency, neurodegeneration, sterility and development (Jackson and Bartek, 2009), combined with the potential tumor suppressive functions of both DDR and chromatin mediated gene silencing (Sulli et al., 2012), the study of the molecular mechanisms by which DDRNAs act is of tremendous interest.

## Conflict of Interest Statement

The author declares that the research was conducted in the absence of any commercial or financial relationships that could be construed as a potential conflict of interest.
